# On the Surgical Treatment of Painful Menstruation

**Published:** 1865-11

**Authors:** J. Marion Sims

**Affiliations:** Bolton-row, May-fair


### Miscellaneous.

### On the Surgical Treatment of Painful Menstruation.

*To the Editor of The Lancet*:

Sir—Please allow me to say a few words in answer to the communication of my friend Dr. Bennet, published in The Lancet, (September No. page 425), "On the Surgical Treatment of Painful Menstruation."

If I have had any misgivings as to the worth of my "clinical notes," they may now be considered at an end. For when so many eminent men step out of the beaten track to discuss their soundness it is almost a guarantee that there is truth at the bottom, the whole of which they cannot at once accept because it does not tally with old preconceived notions.

I am ready to acknowledge my obligations and the debt of gratitude I owe to Dr. Bennet as the father of a correct uterine pathology. In my own country he is *par excellence* the author that we all follow; and his excellent book (like those of Fergusson, Coulson, and Druitt) is found in the library of every well-read medical man. But while we except Dr. Bennet as a light and a guide, we are independant enough *non jurare in verbi magistri*, and we question in many things the soundness of his teachings, and in nothing more than on this very subject of dysmenorrhœa.

My friend thinks that he settled this question many years ago; but I shall be greatly surprised if it is definitely settled in the next generation. The fact is that the pathology of dysmenorrhœa is yet to be written. It is simply a sign or sympton of disease the result of organic change. That organic change may be inflammation, or it may exist independently of it. But whether inflammatory or not, its action is mechanical. I lay it down as an axiom, that there can be no dysmenorrhœa, properly speaking, if the canal of the neck of the womb be straight and large enough to permit the easy passage of the menstrual blood. In other words, that there must be some mechanical obstacle to the egress of the flow at some point between the os internum and the os externum, or throughout the whole cervical canal.

Dr. Bennet says "I have always taught that menstruation may be painful, even acutely painful, from its dawn to its close, without any mischief or impediment existing of any kind whatever." Many years ago I believed all this, simply because Dr. Bennet said so; but now I do not believe in any such doctrine because a large experience has disproved it in every particular. There is no such thing as what Dr. Bennet calls "constitutional dysmenorrhœa." There was a time when we looked upon dropsy as an entity, a disease in itself; but now we know that it is only a symptom of various diseases. It is a sympton of disease of the heart, of the kidneys, of the liver, of the spleen; or it may follow hæmorrhages diarrhœa, &c. So is it with dysmenorrhœa: it is only a symptom of real disease. It may be inflammation of the cervical mucous membrane; retroflection; anteflection; fibroid tumour in one wall of the uterus or the other; contraction of the os internum or os externum; flexures of the canal of the cervix; either acute or gently curved, either at the os internum, at the insertion of the vagina, or extending throughout the whole length of the canal; all of which are but so many mechanical causes of obstructions, which must be recognized and remedied if we expect to cure the dysmenorrhœa. We do not talk of constitutional toothache, of constitutional colic, or of constitutional fractures, or constitutional dislocations; nor should we speak of constitutional dysmenorrhœa. This is but a high-sounding term that means absolutely nothing. The fact is that most of the diseases of the uterus are as purely surgical as are those of the eye, and require the same nice discrimination of the true surgeon; and if we fail to detect the abnormal condition that produces diseased manifestations, whether of sensation or secretion, it is plainly our fault. For all organs the uterus is now most subservient to the laws of physical exploration; and in every case of diseased action, if we cannot map out accurately the pecular condition of the uterus or accompanying it, it is simply because we do not apply our knowledge of those physical laws to its investigation.

But while Dr. Bennet theoretically opposes so strenuously the "mechanical theory of dysmenorrhœa," he acknowledges it in fact, for he says that "dysmenorrhœa showing itself in women who have not had it before, or aggravated when constitutionally present, is, I firmly believe, very much more frequently the result of morbid conditions, of chronic inflammations of the cervix and of the body of the uterus, than of physical obstruction in the cervical canal. When that *obstruction or contraction* exists, it is usually the mere result of the swelling of chronically inflamed or hypertrophied tissues, and disappears without any operation when the inflammation has been removed," &c.

There is no difference of opinion between us here. I have seen just eight cases of this type out of 129 cases dysmenorrhœa.

Again he says: "If very severe, so much so as to cast a gloom over life, either in unmarried or in married women, it is usually connected with inflammatory disease of the uterus, which is also generally the cause of *the narrowing of the cervical canal.*" He very properly tells us to treat this inflammation, and as itsubsides, "generally speaking the cervical passages open, and a natural cure is produced. If they do not thus open, dilatation really becomes necessary, and should be carried out one way or another."

This is at once yielding practically to the whole "mechanical theory of dysmenorrhœa." He acknowledges this "mechanical theory" throughout his entire article, for he advocates the use of sponge-tents in all cases in which he does not recognise a narrowing of the cervical canal by the turgescence of the inflamed mucous membrane. Why should he resort to spong-tents if there in no mechanical barries to overcome? He gives numerous cases illustrating this mechanical treatment, yet he opposes a "mechanical theory."

It seems that Dr. Bennet has for the last twenty years been overcoming mechanical obstructions in the cervix, at one time by Simpson's metrotome and intra-uterine pessaries, with which he "obtained good and permanent results;" but subsequently by sponge-tents.

The only difference between us, seems to be that he opposes the theory of mechanical dysmenorrhœa, but adapts his practice to it, while I permit theory and practice to go hand in hand.

But the real object of the Doctor's paper seems to have been to object to my method of enlarging the os uteri. He talks of "bifurcation" "of slashing operations," "the more severe and serious operations," &c. He seems to have created a windmill out of my operations, and started off to demolish it. But this operation is "not slashing," indeed it is the only one proposed that is not "slashing." It is a simple snip on each side of the os, with a subsequent superficial incision on each side of cervical canal, made with great caution, and which I demonstrated before the Obstetrical Society of London at a recent meeting, showing wherein it was more precise, more surgical, and less apt to be followed by accident than any other operation of the same nature, and that while the incision of the os tincæ was about the same as by Dr. Greenhalgh's metrotome the cutting internally was less. It is not by any means a painful operation. Dr. Bennet has not seen it performed, and draws on his imagination as to its terrors. I well remember how I held my breath in awful suspense when I first read the account of the passage of a freighted train of cars over the suspension bridge at Niagara falls; but when I visited the place six months afterwards, and passed over it in person, I had not the slightest feeling of dread. This was due to the difference between imagination and reality.

I am, Sir, your obedient servant,

J. MARION SIMS, M. D.

Bolton-row, May-fair, July, 1865.

